# Fabrication of Thermal Insulation Bricks Using *Pleurotus florida* Spent Mushroom

**DOI:** 10.3390/ma16144905

**Published:** 2023-07-09

**Authors:** Sally A. Ali, Marwa Kamal Fahmy, Nasser Zouli, Ahmed Abutaleb, Ibrahim M. Maafa, Ayman Yousef, M. M. Ahmed

**Affiliations:** 1 Department of Botany and Microbiology, Faculty of Science, Helwan University, Cairo 11795, Egypt; sally_ali@science.helwan.edu.eg; 2 Department of Architecture, Faculty of Engineering at Mataria, Helwan University, Cairo 11718, Egypt; marwa_fakhry@m-eng.helwan.edu.eg; 3 Department of Chemical Engineering, Faculty of Engineering, Jazan University, Jazan 45142, Saudi Arabia; azabutaleb@jazanu.edu.sa (A.A.); imoaafa@jazanu.edu.sa (I.M.M.); 4 Department of Mathematics and Physics Engineering, Faculty of Engineering at Mataria, Helwan University, Cairo 11718, Egypt; marwa_elnagar77@yahoo.com

**Keywords:** lightweight bricks, spent mushroom materials, bio-based materials, thermal insulation, energy efficiency buildings

## Abstract

This study explores the potential for making lightweight bricks via the use of dry, pulverized spent mushroom materials (SMM) as a thermal insulator. There are five distinct replacement proportions of SMM that are used, and they range from 0% to 15% of the weight of the clay. The firing of the fabricated bricks at temperatures of 700, 800, and 900 °C led to the development of pores on the interior surface of the bricks as a consequence of the decomposition of SMM. The impact of SMM on the physicomechanical characteristics of fabricated bricks is assessed based on standard codes. Compressive strength, bulk density, and thermal conductivity decreased as the SMM content increased, reaching up to 8.7 MPa, 1420 kg/m^3^, and 0.29 W/mK at 900 °C and 15% substitution percentage. However, cold water absorption, boiling water absorption, linear drying shrinkage, linear firing shrinkage, and apparent porosity increased with the increase in SMM, reaching 23.6%, 25.3%, and 36.6% at 900 °C and 15% substitution percentage. In the study simulation model, there was a significant improvement in energy consumption, which reached an overall reduction of 29.23% and 21.49% in Cario and Jazan cities, respectively.

## 1. Introduction

Due to the overexploitation of numerous natural resources, there is a lack of natural resources in the world for manufacturing conventional bricks [1]. Huge amounts of raw materials are consumed in the brick industry [2,3]. To overcome this issue, several attempts have been made to incorporate different waste materials into the brick-making process, including natural fibers, textile laundry wastewater sludge, foundry sand, granite sawing waste, perlite, processed waste tea, sewage sludge, structural glass waste, fly ash, sugar cane bagasse ash, organic residue, steel dust, bottom ash, rice husk ash, silica fume, and municipal solid waste incineration fly ash [4,5,6,7]. An innovative biotechnological method for recycling lignocellulosic waste involves growing oyster mushrooms. Mushroom substrates have a limited ability to condition and fertilize soil due to their high salt, nutrient, and alkaline contents [8]. Cellulosic substrates, including cotton wastes, maize cob wastes, bean straws, crushed bagasse, molasses wastes, coffee husks, paper wastes, industrial cardboard wastes, tree sawdust, and rice straws can be used to grow mushrooms. The majority of commercially grown Agaricus mushrooms are grown on straw or hay substrates. Rice straw mushrooms (RSM) are regarded as a nutritious food (USITC 2010) [9,10]. Growing edible mushrooms meets the dual goals of treating rice straw, which acts as a substrate for the production of food (mushrooms), and as a source of food for the mushroom-spent bedding. One of the biggest environmental problems in the mushroom production process that utilizes rice straw as soil is the vast amounts of spent mushroom material (SMM) generated as solid waste by-products—1 kg of mushrooms produces around 5 kg of solid waste [11]. SMM waste is frequently disposed in landfills [12]. About 27 billion kg of cultivated mushrooms were produced worldwide in 2012 [13]. Governments and researchers are examining the potential uses of these leftover materials as a result of careful environmental management of this by-product of mushroom production. Today, around 50% of the population lives in cities, using a lot of energy resources and emitting more than 70% of the world’s carbon emissions. This is mainly because of urban development, which has been identified as the cause of issues with sustainability [14]. One approach for addressing this is using sustainable materials such as SMM in the production of construction materials. This has some beneficial effects on the environment and the economy and, in addition to optimizing the thermal performance of the urban block form, is necessary for the management of daily wastes that cause significant environmental problems [15]. Beyond the context of the surrounding, the building’s shape and the windows’ parameters have an impact on energy consumption [16]. Using sustainable materials with thermally isolated behavior is considered the most effective parameter for achieving sustainability. Inefficient thermal materials, which cause high energy consumption, have environmental and economic consequences during the building’s life [17,18]. Hence, avoiding or even reducing as much thermal gain as possible in buildings decreases the internal temperature and improves the indoor thermal quality, thus directly decreasing the need for air conditioning, resulting in a decrease in the energy consumed in the building and the city. The main distinguishing objectives and novelty of this study are managing waste materials by producing lightweight bricks made by replacing clay with SMM materials in various ratios that meet the obligatory values of the physicomechanical characteristics assigned by standards. It was also critical for manufacturing lightweight bricks with effective thermal insulation for controlling energy usage. These bio-based, lightweight bricks can both help with trash disposal and resource conservation because they are made from rice straw waste that has undergone the mushroom biodegradation process. Thus, it makes economic and environmental sense to utilize SMM as a clay body addition. Table S1 contains the physicomechanical and thermal characteristics of bricks substituted with different waste materials.

## 2. Experimental

### 2.1. Materials and Methodology

Clay was obtained from Aswan, Egypt, and spent mushroom materials (SMM) was grown in the laboratory. *Pleurotus florida* spawn (culture) was obtained from the Agricultural Research Centre, Ministry of Agriculture, Giza, Egypt.

### 2.2. Preparation of Spent Mushroom Materials from Cultivated Pleurotus florida

**Figure 1:**
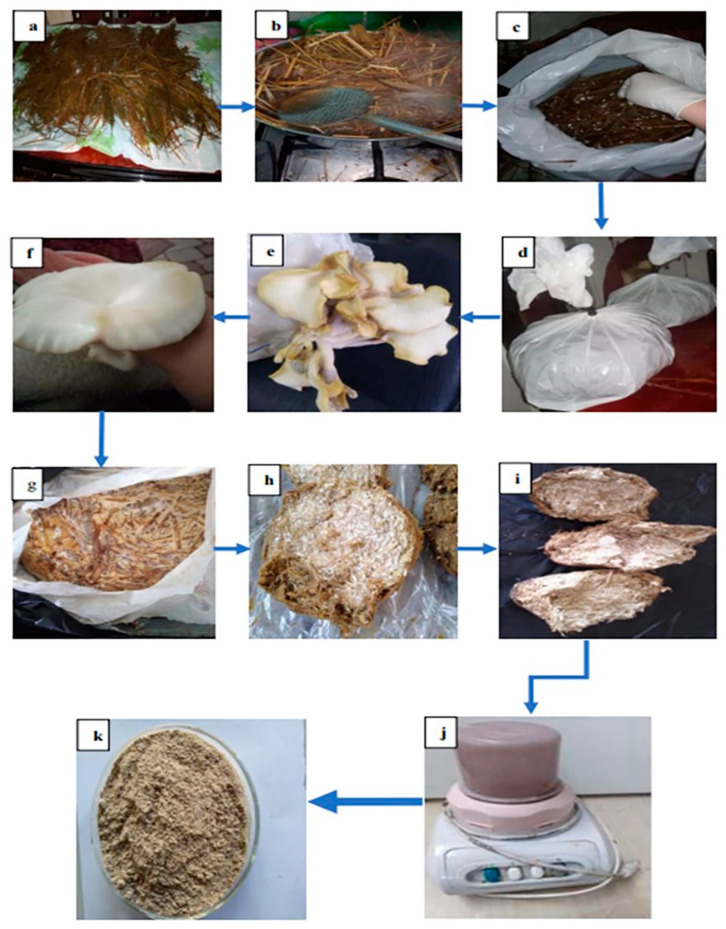
Preparation of SMM from cultivated *Pleurotus florida* (**a**) chopped rice straws, (**b**) soaked rice straw, (**c**) inoculation of rice straws with 50 g *Pleurotus florida* spawn, (**d**) sealed, inoculated, and sterilized bags, (**e**) fruitful body development, (**f**) first harvest, (**g**) formation of SMM, (**h**) SMM after autoclaving, (**i**) SMM after heat drying, (**j**) A pulverizer, and (**k**) powdered spent mushroom materials in a petri dish.

Spent mushroom materials (SMM) of *Pleurotus florida*—also known as the oyster mushroom or the white oyster mushroom—were prepared after cultivation (Figure 1). The SMM was prepared in the laboratory to show the process of mushroom growth. The preparation was carried out following the protocol by Stoknes et al. [19], with slight modifications as follows: To ensure that water completely soaked through the rice straws, the straws were cut into 20–30 cm lengths, soaked in water that was three times as heavy as the straw, left to soak for 24 h, and then air dried. After soaking, the rice straws were placed and sealed in sterile plastic bags. The bags were subjected to a 15-min sterilization process at 121 °C and then cooled at room temperature for 24 h. Each bag was filled with 50 g of *Pleurotus florida* spawn (culture) that had already been prepared and cooled; the bag was then sealed. The inoculated rice straw was incubated at 25 °C with light requirements of 1000–1500 (2000 lux) for not less than 30 days, until fruit body development. The first harvest was carried out after 30–40 days, and the yield during the first harvest was 250 g. The raw materials of this edible fungus, as well as the cultivation and harvesting conditions for spent mushroom material preparation, are shown in Figure 1a–k. After autoclaving for 20 min and drying at 60 °C for 48 h, each piece of residual *Pleurotus florida* spent mushroom material was crushed using a pulverizer (Elaraby, Cairo, Egypt, MX 900/2). Each substrate’s powder was then separated into two parts using a sieve (through a mesh with a 2-mm pore size sieve). The sieving process is performed to separate the impurities and ungrounded particles, and only the finer part was used for the experiments.

### 2.3. Characterization of Clay and Spent Mushroom Materials

In this investigation, X-ray fluorescence (XRF) was used to assess the chemical composition of Aswan clay and SMM after they had been ground into powder using an AXIOS, Panalytical 2005 (Malvern Panalytical, Malvern, UK), and a wavelength dispersive (WD) XRF sequential spectrometer (Malvern Panalytical, Malvern, UK). By utilizing the Brukur (Billerica, MA, USA) D8 advanced computerized X-ray diffractometer apparatus with monochromatized CuK radiation operated at 40 kV and 40 mA, it was possible to establish the mineralogical composition of the raw material. The acquired SMM powder was used directly without further processing. The ASTM C136 standard sieve analytical approach was used to calculate the mean particle size [20].

### 2.4. Preparation and Testing of Samples

**Figure 2:**
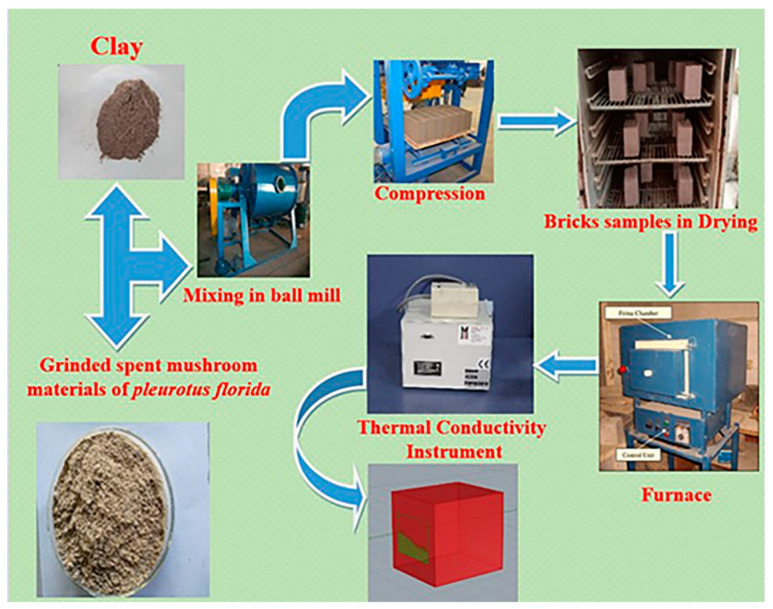
Schematic of the brick production process.

**Figure 3:**
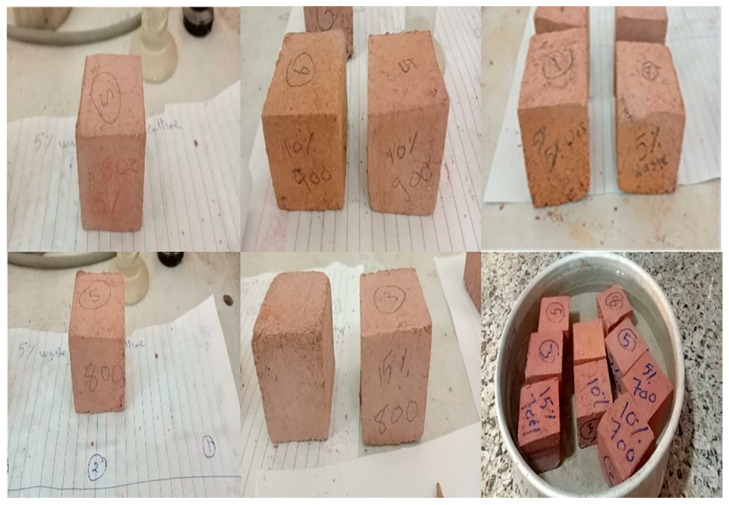
Photo-image of the fabricated bricks.

The clay was substituted with six different percentages of SMM ranging from 0% to 15% by weight. In order to obtain a homogenous mixture, the raw components were stirred at a speed of 50 rpm for 10 min. The pastes were produced by adding 18 wt.% of water to each of the aforementioned mixtures to get the desired consistency. They were molded into steel cube molds (50 × 50 × 50 mm^3^) and compressed under 10 MPa pressure. The formed samples were left out in the open for a period of 12 h where they were exposed to direct sunlight. The samples were then de-molded and dried overnight in an oven at a temperature of 120 °C. In the final stage, the samples were sintered at three different temperatures: 700, 800, and 900 °C. The samples were kept in the furnace for a total of 4 h at each temperature. Figure 2 shows the preparation procedure for the bricks. The percentages of linear drying shrinkage for the dried samples were measured. Additionally, linear firing shrinkage, cold and boiling water absorption, apparent porosity, bulk density, compressive strength, and thermal conductivity tests were used to analyze the characteristics of fired samples based on ASTM standard codes and similar to our previous work [3]. The samples were tested in triplicate, and the average value of the parameter under investigation was computed. Figure 3 shows the photo-image of the fabricated bricks.

### 2.5. Raw Material Characterization

**Figure 4:**
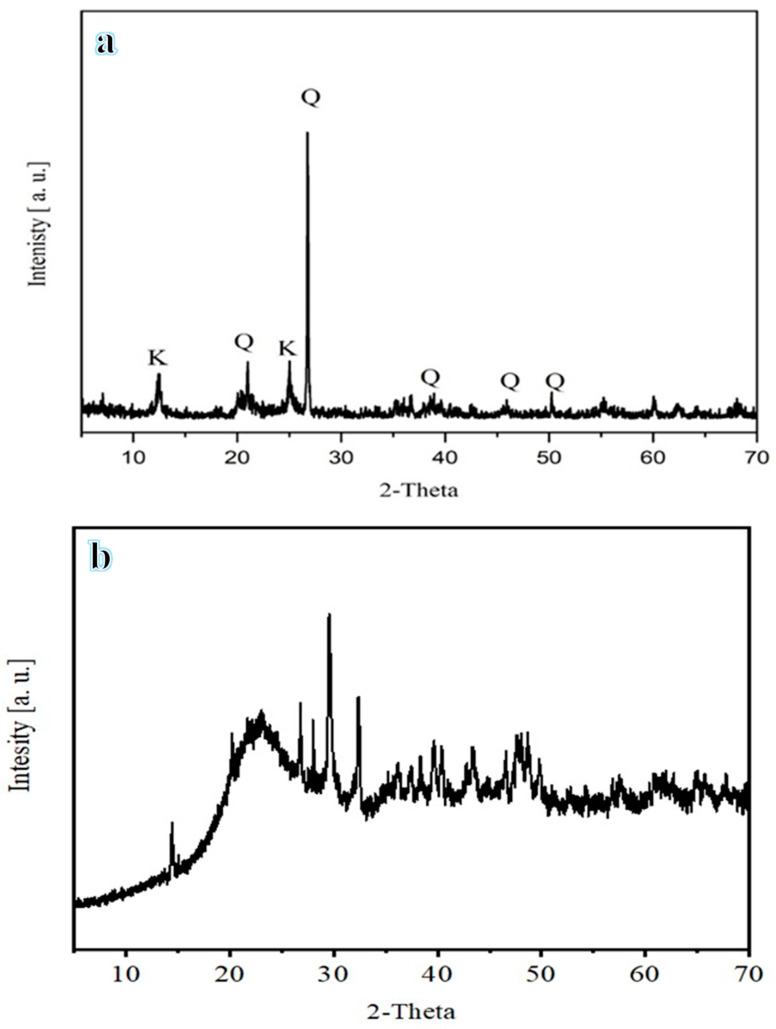
XRD analysis of clay (**a**) and SMM (**b**).

Figure 4 shows the XRD of the raw materials. As seen in the figure, silica and alumina were the two primary compounds present in the clay (Figure 4a), while SMM is composed of sodium aluminum silicate, davidsmithite, trinepheline, sodium aluminum silicate hydroxide, and wollastonite (Figure 4b). Table 1 displays the XRF analysis of the raw materials. The results indicate that SiO_2_ and Al_2_O_3_ are the major compounds in clay, with compositions of between 20% and 50% of SiO_2_ and between 10% and 20% of Al_2_O_3_, which were within the recommended ranges [68, 69].

**Figure 5:**
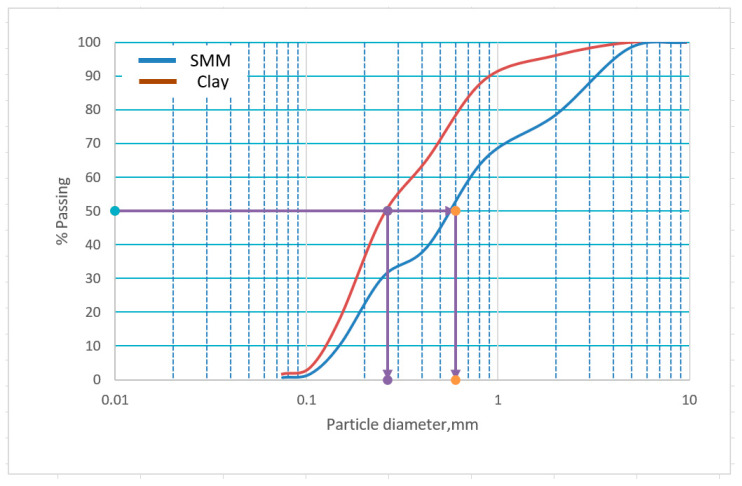
Distribution of the raw materials’ particle sizes.

SiO_2_ and Al_2_O_3_ were present in lower quantities in SMM. However, it contained more calcium oxide (CaO) and potassium oxide (K_2_O) than clay. Furthermore, its high organic content is thought to be the cause of its significant loss on ignition (LOI) [21]. Kaolin dehydroxylation is considered the primary cause of the LOI and raising thermal conductivity [22]. Figure 5 displays the particle size distribution of the raw materials. This graph demonstrates the differences in their median sizes (D50), which equal 0.26 mm for clay and 0.6 mm for SMM, demonstrating that clay is finer than SMM.

### 2.6. Simulation Procedure

**Figure 6:**
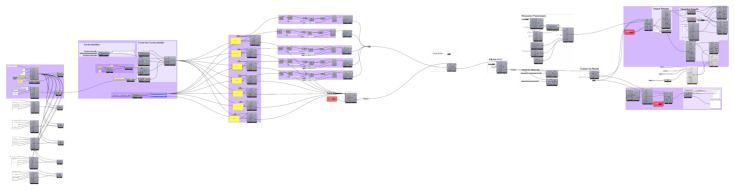
Definition made in Grasshopper.

The study optimized the building design, aiming to achieve optimum thermal performance. First, the model was built in Grasshopper (Figure 6). Then, the thermal model was written in Grasshopper using Honeybee, ladybug, and climate studio (Grasshopper plugins). The plugins work as engines for EnergyPlus, Radiance, Daysim, and OpenStudio [11]. Different materials were identified and inserted into the model as exterior wall materials. The materials were made from Energy Plus 23.1.0 materials. Optimization began with simulation of different types of bricks in a room as a case study. The simulation included studying cooling loads and CO_2_ emissions.

### 2.7. Description of the Study Model

**Figure 7:**
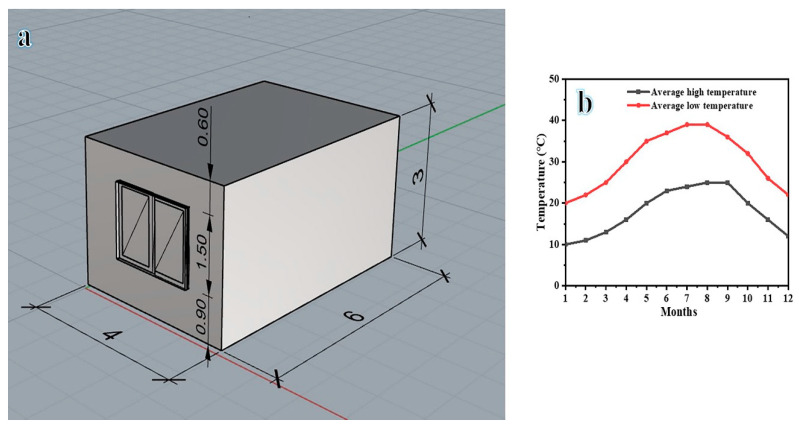
Study model (**a**) and average temperature (**b**).

The case study consisted of one unit with dimensions of 4.0 m width, 6.0 m length, and 3.0 m height, located in Cairo, Egypt (Figure 7a). Figure 7b shows the average temperature in Cairo, Egypt. The impact of the new wall section made of different fabricated bricks was compared with the base case. The energy saved using different fabricated bricks was calculated. The exterior walls were 0.25 m thick and contained three layers (plaster–brick–plaster). The material used was EnergyPlus Material. For the HVAC system, the Honeybee plugin acted as an engine for EnergyPlus, and the air conditioning system was set to 24 °C. The building operated 24 h/day as it is a residential unit. Energy loads were simulated using Honeybee as an engine for Radiance, Daysim, OpenStudio, and EnergyPlus, and energy performance was evaluated through cooling loads. The building’s annual energy consumption (kWh/m^2^) was computed for easier comparison between various materials.

## 3. Results and Discussion

### 3.1. Linear Shrinkage

**Figure 8:**
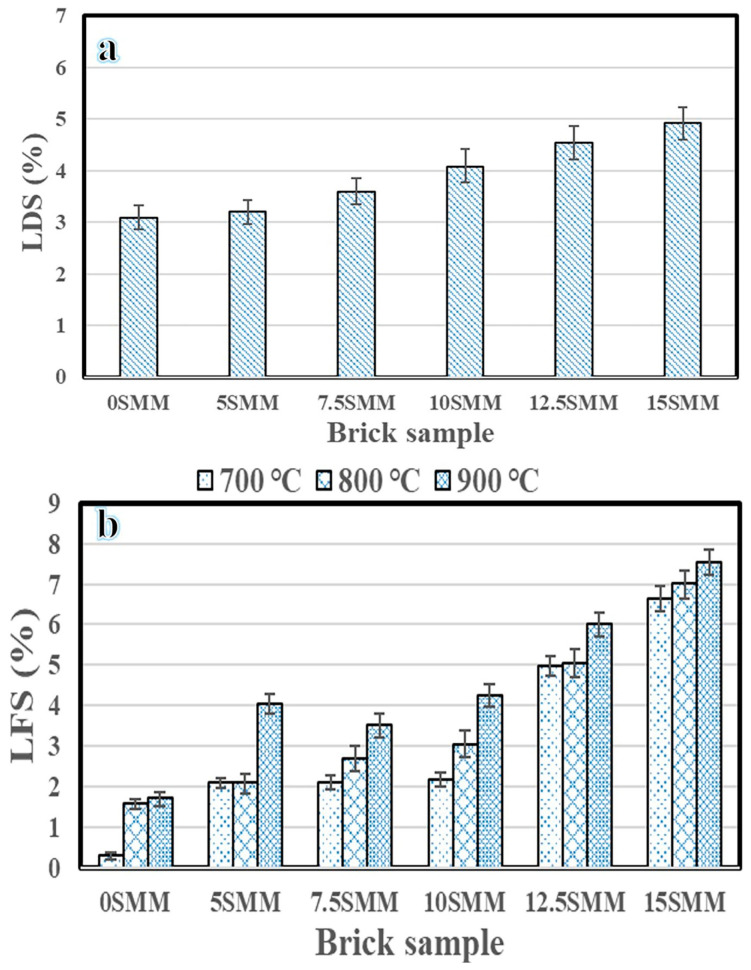
Linear drying shrinkage (**a**) and firing shrinkage (**b**).

Due to water loss, the clay particles became compact as a result of shrinkage. The increase in the SMM content led to an increase in linear drying shrinkage (Figure 8a). Compared with the control sample, which displayed a linear drying shrinkage of 3.17%, the sample composed of 15% SMM had a maximum linear drying shrinkage of 4.9%. This effect can be attributed to the reduction in quartz content, which helped to increase the volumetric stability of the mixture [23]. The degree of densification during a fire is often determined in part by shrinkage. SMM particles fuse during sintering at high temperatures, resulting in more closely packed particles that improve linear firing shrinkage. Firing temperature is a crucial factor influencing the degree of shrinkage, and large shrinkage could be problematic since it might result in fissures and dimensional flaws. To reduce shrinkage, the firing temperature must be managed during the sintering process. It is worth mentioning that the mechanical performance of the bricks can be maintained after sintering if the linear firing shrinkage is less than 8%. As shown in Figure 8b, firing shrinkage increases with the increase in the amount of SMM and the firing temperature. The findings revealed that shrinkage in the fired clay brick samples ranged from 6.6% to 7.6% with the addition of 15% SMM, while firing shrinkage in the control fired clay bricks ranged from 0.29% to 1.7% without the addition of any SM; thus, shrinkage is influenced by the concentration of the added SMM. All brick samples’ shrinkage after firing fell within the permitted range for commercial manufacturing, as stated in ASTM C326 [24]. However, the linear shrinkage of all the evaluated additions was under 8%. The sample composed of 15 wt.% SMM showed 7.6% linear firing shrinkage at 900 °C.

### 3.2. Bulk Density

**Figure 9:**
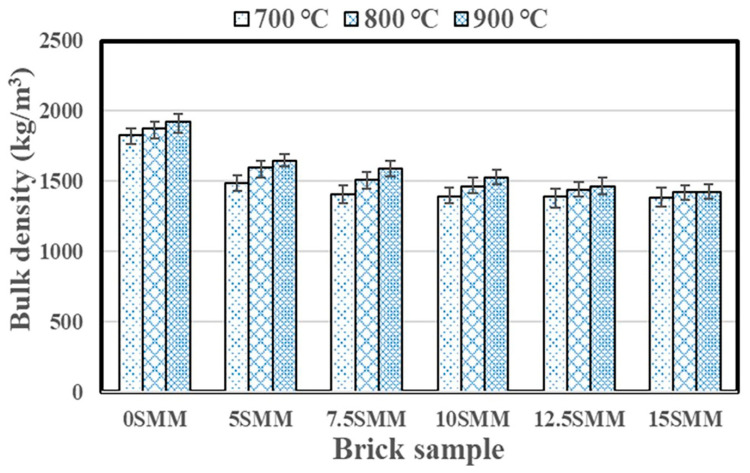
Average values of the bulk density of the samples.

The specific gravity of the raw material source, the manufacturing process, and the firing temperature can all influence the bulk density of fired clay bricks. Figure 9 shows the influence of SMM and firing temperature on the bulk density of the fired clay bricks. The findings indicate that the maximum replacement percentage (15% SMM) produced the lowest bulk density of 1385.2, 1420.8, and 1419.99 kg/m^3^ when the samples were subjected to firing temperatures of 700, 800, and 900 °C, respectively. Under the same firing temperatures of 700, 800, and 900 °C, the bulk density was 1828.7, 1874.2, and 1922 kg/m^3^, respectively, for the brick composed of 0% SMM. Accordingly, the bulk density reduced as SMM content increased, possibly due to the increased formation of pores that occur as a result of the degradation of carbonaceous matter in SMM during the sintering process [25]. On the other hand, bulk density increased slightly as the firing temperature increased at each percentage of SMM, possibly due to the verification or higher consolidation between body particles during the sintering process. Based on the results of the bulk density measurement, the brick can be categorized as lightweight if it has a density lower than 1680 kg/m^3^ in accordance with the requirements of the standard ASTM C90 [26,27]. The bricks met the lightweight standard at all SMM percentages analyzed.

### 3.3. Water Absorption

**Figure 10:**
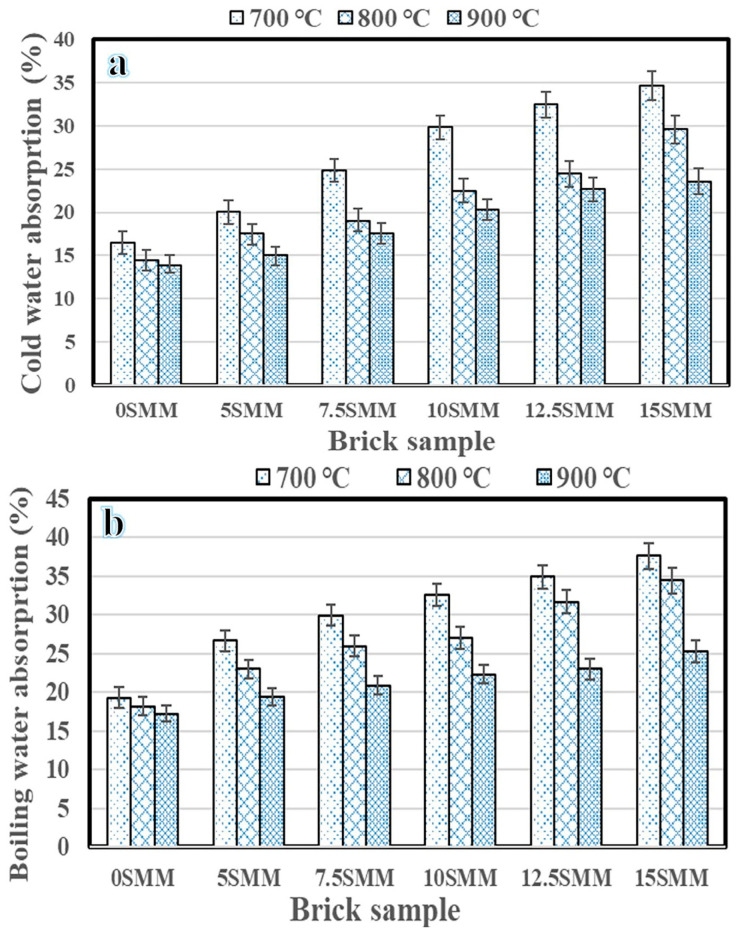
Cold water absorption (**a**) and boiling water absorption (**b**).

The findings of water absorption by fabricated bricks at 700, 800, and 900 °C, and the clay replacement percentages varying from 0% to 15% SMM are shown in Figure 10. Brick durability can be assessed by measuring its ability to absorb water or by its physical characteristics. As shown in the figure, water absorption by bricks is linearly correlated with the percentage of SMM present, indicating that SMM serves as a pore-generating agent. When clay was substituted with 15 wt.% of SMM, the rate of absorption of cold water rose to 34.7%, 29.6%, and 23.6% at 700, 800, and 900 °C, respectively, compared with 16.4%, 14.5%, and 13.9% at 0% SMM substitution (Figure 10a). On the other hand, boiling water absorption was 37.6%, 34.5%, and 25.3% when 15% SMM was used as a substitute for clay, up from 19.3%, 18.2%, and 17.2% at 700, 800, and 900 °C, respectively, when 0% SMM substitution was used (Figure 10b). Furthermore, the bricks demonstrated the lowest water absorption percentage and pore volume at 900 °C in accordance with the requirements of the standard ASTM C62 [6,28], likely due to the increase in the formation of a glassy phase at high firing temperatures. In general, water absorption increased with the increase in the SMM in the mixtures and decreased with increasing firing temperature. Brick samples with high water absorbency had greater total porosity, improving the insulating characteristics of the clay bricks.

### 3.4. Apparent Porosity

**Figure 11:**
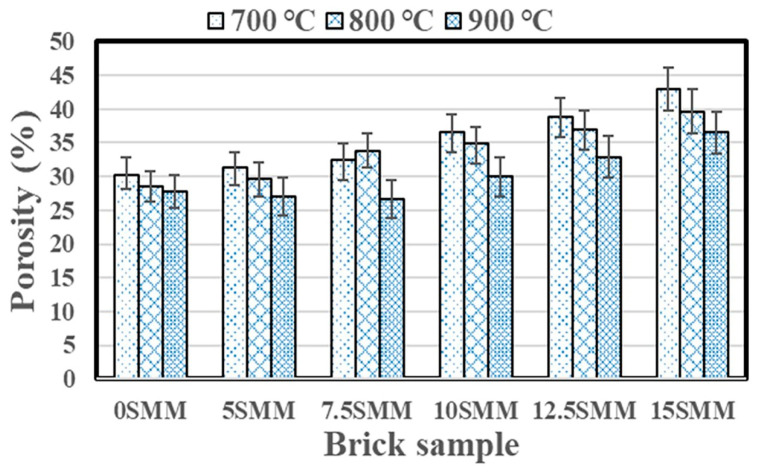
Apparent porosity results.

Figure 11 illustrates the findings of apparent porosity tests conducted on brick samples burnt at temperatures of 700, 800, and 900 °C using SMM clay-replacement percentages varying from 0 to 15%. It was noted that the brick samples containing SMM had more porosity than the control bricks at different temperatures. This could be due to the gases resulting from the decomposition of carbonaceous materials and the fact that SMM has a higher LOI than clay [23]. According to Sutcu and Akkurt [29], an increase in overall porosity of over 50% is effective at improving the insulating qualities of fired clay bricks. Overall, total porosity increased almost linearly as the percentage of SMM rise, demonstrating the effectiveness of SMM as a pore-forming agent. The results demonstrated an increase in porosity of 29.6, 27.9, and 24.2% when clay was substituted with 15 wt.% SMM at firing temperatures of 700, 800, and 900 °C, respectively. Brick samples fired at 900 °C exhibited the lowest porosity, likely due to the formation of the glassy phase at high temperatures. Although the formation of pores in the brick structure is the cause of decreased compressive strength, higher water absorption, and total porosity, the increased porosity has a positive impact on the performance of bricks because these pores make it easier for water vapor to move inside the brick skeleton, thereby preventing cracks from spreading, especially in humid climate zones [30].

### 3.5. Compressive Strength

**Figure 12:**
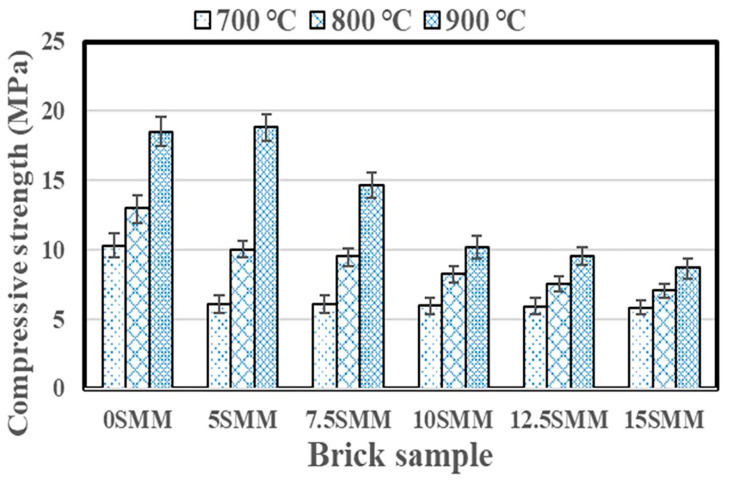
Compressive strength results.

Figure 12 indicates the findings of compression tests conducted on fired brick specimens. The compressive strength of the samples was determined using the equation below; the burnt samples were tested by applying a perpendicular force that is consistent with ISO 9652 [31].

(1) $$C=\frac{P}{A},\left(\frac{N}{{\mathrm{mm}}^{2}}\right)$$

The variable C denotes the compressive strength of fired samples, measured in units of N/mm^2^ (MPa). The variable “P” denotes the maximum load, while the variable “A” represents the average cross-sectional area of the fired samples. The mean compressive strength of the specimen was determined by computing the average of the compressive strengths of three distinct brick specimens. Based on the findings in the figure, the addition of SMM and firing temperature had a significant impact on the compressive strength of fired clay bricks. While the addition of SMM did enhance some physical features, it had a detrimental effect on some mechanical characteristics. After sintering the fabricated bricks at 700 °C, the compressive strength of the brick specimens decreased from 10.23 MPa to 6.06 MPa for 5 SMM, 6.04 MPa for 7.5 SMM, 6 MPa for 10 SMM, 5.89 MPa for 12.5 SMM, and 5.8 MPa for 15 SMM. Due to the significant number of pores and cavities produced by the decomposition and combustion of organic matter, the integration of SMM, as anticipated, decreased the compressive strength [32]. The greater the pore size due to the decomposition of SMM, the smaller the exposed area of the compressive-resistant brick section, suggesting that this may be the cause of the drop in compressive strength. However, increasing the brick firing temperature to 800 °C increased the compressive strength to 12.9 MPa for the 0 SMM sample, 10 MPa for the 5 SMM sample, 9.5 MPa for the 7.5 SMM sample, 8.2 MPa for the 10 SMM sample, 7.6 MPa for the 12.5 SMM sample, and 7 MPa for the 15 SMM sample. In addition, increasing the brick firing temperature to 900 °C substantially improved the compressive strengths of the 0 SMM, 5 SMM, 7.5 SMM, 10 SMM, 12.5 SMM, and 15 SMM samples to 18.4 MPa, 18.8 MPa, 14.6 MPa, 10.13 MPa, 9.5 MPa, and 8.6 MPa, respectively. By sealing off internal pores, raising the firing temperature significantly accelerated verification and improved the development of strength. This is consistent with other studies that found that bricks produced at higher firing temperatures have higher densities and mechanical strengths [33,34]. Many studies [19] have shown that when firing temperatures rise, the bulk density of the brick framework increases, leading to greater compressive strength in the brick. The compressive strength requirements for non-load-bearing clay bricks are set at 3.5 N/mm^2^ by the IS 1077-1992 standards [35]. Therefore, bricks having 15% SMM sintered at 800 or 900 °C still meet the required standards. The ASTM C62 [28] minimum suggested a compressive strength value of 8.6 MPa, which is consistent with brick samples fired at 800 and 900 °C. Furthermore, bricks containing 5% SMM and fired at 900 °C produced the highest compressive strength of 18.9 MPa.

### 3.6. Thermal Conductivity

**Figure 13:**
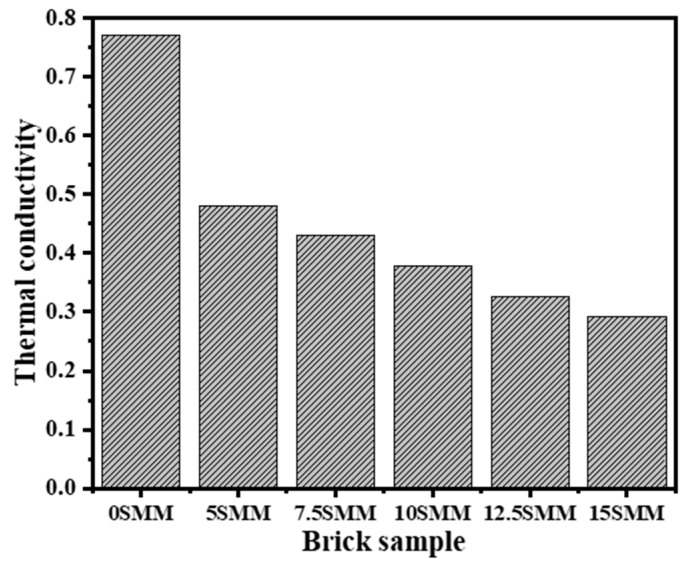
Thermal conductivity results.

Figure 13 shows the variance in thermal conductivity of bricks fired at 900 °C as a function of the SMM content. As shown in the figure, thermal conductivity reduced as the SMM content increased. The brick-free SMM had the greatest thermal conductivity (0.77 W/mK), with the brick being denser than other formulations; on the other hand, bricks composed of 15% SMM had the lowest thermal conductivity (0.293 W/mK). As is known, heat is transferred through solid materials by free electrons and/or lattice vibration waves (phonons). In ceramic materials, thermal conduction results from phonons. The presence of pores in the structure of ceramic materials facilitates phonon scattering, which may reduce the thermal conductivity of ceramic materials [36]. The bricks fired at 900 °C, including 5 SMM, 10 SMM, 12.5 SMM, and 15 SMM, showed significant reductions of 0.481, 0.43, 0.37, 0.32, and 0.29 (W/mK), respectively, in thermal conductivity compared with 0.77 (W/mK) of 0 SMM. The development of pores during sintering as a result of the production of gases that arise from the decomposition of carbonate materials and the high LOI in the SMM may be a possible explanation for the decrease in heat conductivity that occurs with the increase in the proportion of SMM in the material.

### 3.7. SEM Microstructure Analysis

**Figure 14:**
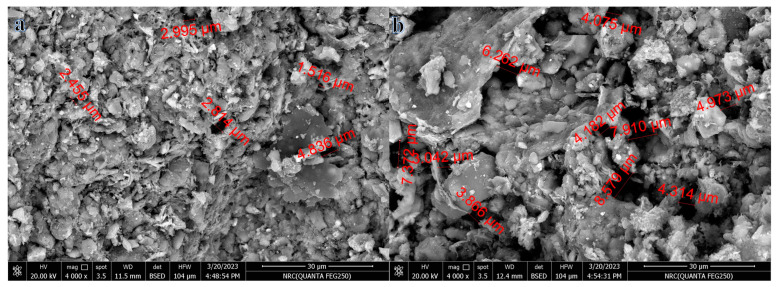
SEM images of 5% (**a**) and 15% (**b**) SMM bricks fired at 900 °C.

Figure 14 shows the microstructural characteristics of bricks that were burned at 900 °C and contained either 5% or 15% SMM. It is plain to see that the bricks that were infused with SMM produced a sizeable number of micropores, all of which were dispersed throughout the matrix of the hardened bricks. The pores that were visible took on a number of different shapes, including spheres and ovals. When the SMM percentage was increased, both the number of micropores and their diameters also increased. The average pore size increased from 2.92 to 5.71 µm when the percentage of SMM increased from 5% to 15%. This helps to explain the decrease in thermal conductivity, compressive strength, and bulk density, as well as the rise in water absorption and apparent porosity.

### 3.8. Estimation of Annual Energy Consumption for Cooling

**Figure 15:**
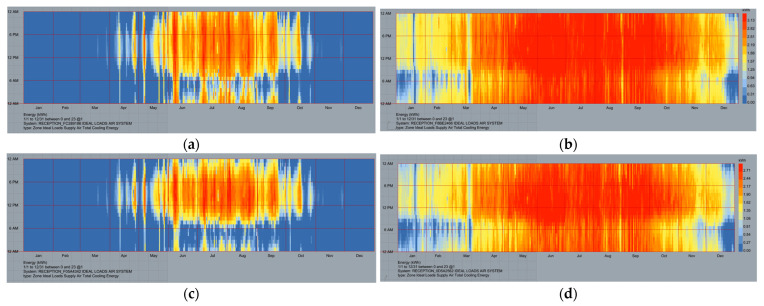
Monthly required cooling load for 0 SMM in Cairo (**a**) and Jazan (**b**) and for 15 SMM in Cairo (**c**) and Jazan (**d**).

**Figure 16:**
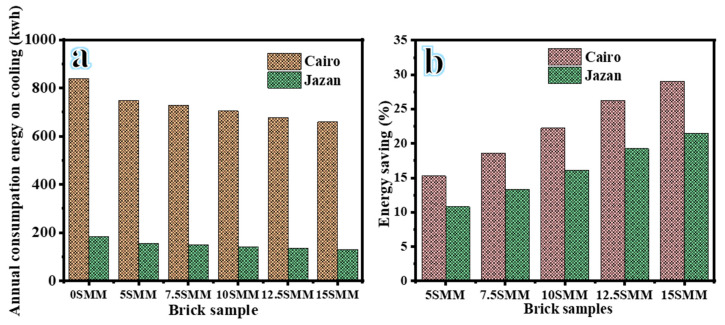
Annual energy consumed for cooling (**a**) and percentage of energy saved (**b**) using different percentages of SMM.

The effect of bricks with different SMM proportions on a case study’s cooling demands was evaluated using Grasshopper plugins Rhin 7 thermal simulation software. The application starts by simulating a room as a basic case to identify the optimal SMM ratio in terms of energy efficiency. As can be seen in Figure 15, the monthly variation in energy use was largest during the summer months of April through October. In addition, traditional bricks consumed the highest energy for cooling (Figure 15a,b) in both Cairo and Jazan cities. Samples with 15 SMM bricks used substantially less energy for cooling than their standard counterparts (Figure 15c,d) in Cairo and Jazan cities. The highest monthly average for cooling energy used occurred in July, the warmest month of the year; this was particularly true in the mornings. Figure 16 shows the simulation results, which indicate that different SMM brick compositions affect cooling energy consumption. The replacement of standard bricks with a 15 SMM brick sample reduced the building’s cooling needs by 129.514 kWh, which is approximately 29.23%, compared with the 0 SMM (183 kWh) bricks (Figure 16a). The total amount of energy that was utilized for cooling reduced by 134.606 kWh, which is an improvement of 26.44%, when 12.5 SMM was used. Furthermore, cooling energy consumption can be reduced by 141.898 kWh with 10 SMM, 148.611 kWh with 7.5 SMM, and 154.63 kWh with 5 SMM, representing improvement rates of 22.46%, 18.79%, and 15.50%, respectively, (Figure 16b). This significantly reduces the building’s energy consumption, as space cooling accounts for 37–42% of its overall energy requirement [37,38]. The suggested manufactured brick samples were examined for thermal and energy efficiency in the city of Jazan in the Kingdom of Saudi Arabia (Figure 16a,b), which has a climate characterized by hot, arid desert conditions. The sample bricks were compared with a standard brick for the purpose of establishing a reference point. The hypothesis posits that a model identical to that implemented in Cairo should be utilized in Jazan. Using 15 SMM resulted in a reduction in energy consumption of 21.49%. Utilization of clay bricks derived from SMM exhibited promising prospects of enhancing thermal efficiency and energy conservation and reducing the carbon footprint in arid and high-temperature settings.

### 3.9. Influence of the Proposed Fabricated Bricks on CO_2_ Emissions

**Figure 17:**
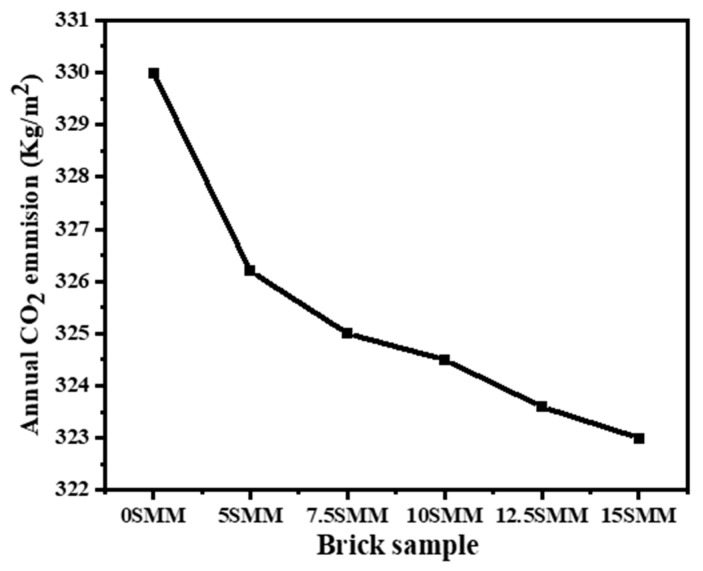
Annual CO_2_ emission of the fabricated bricks.

Embodied CO_2_ has become an increasingly significant contributor to the entire life-cycle of construction in the last few decades. This is the case despite the fact that developments in technology have led to a decrease in operational CO_2_ emissions. The main sources of operational CO_2_ emissions are HVAC and lighting; the major sources of embodied CO_2_ emissions are construction materials [39]. The results of this study suggest a link between CO_2_ emissions from building operations and the increasing prevalence of air conditioning in hot climates. The construction industry likely contributes to the rising atmospheric concentrations of embodied and operational CO_2_. Construction materials have been the subject of extensive studies and developments. As a result, buildings’ thermal performance has improved, the amount of energy required to keep them cool has decreased, and CO_2_ emissions have decreased. The results of this research suggest that SMM bricks may benefit building occupants’ health by decreasing their dependence on air conditioning and slowing the rate at which heat travels through walls. As a result, indoor temperatures will be more pleasant. In addition, it is possible that cutting down CO_2_ emissions would be possible thanks to the utilization of SMM in bricks. Figure 17 shows the massive amounts of CO_2_ emissions produced in order to provide cooling. It was demonstrated that using 5 SMM might result in an annual decrease of 2.2% in CO_2_ emissions.

## 4. Conclusions

The following are inferences drawn from the study’s practical findings:

- The use of SMM as a pore-forming material allows for the production of fired bricks with low heat transmission and sufficient compressive strength. Linear firing shrinkage is adversely affected by the replacement of SMM waste. When the operating temperature is raised, there is a corresponding rise in firing shrinkage; the bricks produced at 900 °C with 15% SMM showed a major increase (7.6%) in firing shrinkage.
- The results demonstrate that increasing the quantity of SMM as a substitute for clay leads to a loss in compressive strength. The most modest compressive strength, 8.7 MPa at 900 °C, was achieved at the highest SMM % and may be adequate to fulfill the standards for load-bearing blocks in ordinary constructions in accordance with ASTM C62 [28].
- The bulk densities of all samples reduced as the percentage of SMM added as a replacement for clay increased, and samples containing SMM were less dense compared with the control sample.
- Because of the correlation between SMM and water absorption and porosity, decreased compressive strength, bulk density, and thermal conductivity are observed when SMM is increased. Regardless of firing temperature, higher SMM concentrations result in lower thermal conductivity by the brick specimens. When burnt at 900 °C, brick samples containing 15 wt.% SMM exhibited the smallest thermal conductivity (0.29 W/mK).
- SEM analysis of the SMM bricks indicated that the number and diameters of micropores increased as the SMM ratio increased.
- Thermal efficiency is maximized in bricks containing 15% SMM. In the case study provided, a 2.2% decrease in CO_2_ emissions and a 29.23% decrease in energy consumption were achieved.

## Figures and Tables

**Table 1:** Chemical composition of raw materials.

Composition	Clay wt. (%)	SMM wt. (%)
Al_2_O_3_	32.906	0.07
SiO_2_	48.931	30.2
Na_2_O	0.094	0.38
K_2_O	0.014	0.66
CaO	0.505	1.58
MgO	0.09	--
TiO_2_	5.918	--
Fe_2_O_3_	1.193	0.1
SO_3_	0.291	--
F	--	--
Cl	0.011	--
Cr_2_O_3_	0.138	--
ZrO_2_	0.465	--
LOI	9.2	32
TOTAL	99.756	64.99

## Data Availability

The data presented in this study are available from the corresponding authors upon reasonable request.

## Supplementary Materials

The following supporting information can be downloaded at: https://www.mdpi.com/article/10.3390/ma16144905/s1, Table S1: Demonstrates physco-mechanical and thermal characteristics of bricks substituted with different waste material. References [40,41,42,43,44,45,46,47,48,49,50,51,52] are cited in the supplementary materials.
