# Supplementary material for: Fabrication of Thermal Insulation Bricks Using Pleurotus florida Spent Mushroom
Source: Materials (Basel). 2023 Jul 9;16(14):4905. doi: 10.3390/ma16144905 (PMC10381914; doi:10.3390/ma16144905)
Supplement: Supplementary file 1 [file materials-16-04905-s001.zip › materials-2452264-supplementary.pdf]

Fabrication of Thermal Insulated Bricks-Based on partial replacement of *pleurotus florida* Spent Mushroom Materials as Energy Saving Building Material

Sally A. Ali[1], Marwa Kamal Fahmy[2], Nasser Zouli[3,*], Ahmed Abutaleb[3], Ibrahim M. Maafa[3], Ayman Yousef[3,4,*], M. M. Ahmed[4]

[1]Botany and Microbiology Department, Faculty of Science, Helwan University, Cairo 11795, Egypt.

[2]Architecture Department, Faculty of Engineering at Mataria, Helwan University, Cairo 11718, Egypt.

[3]Chemical Engineering Department, Faculty of Engineering, Jazan University, Jazan 45142, Saudi Arabia.

[4]Mathematics and Physics Engineering Department, Faculty of Engineering at Mataria, Helwan University, Cairo 11718, Egypt.

Corresponding authors:

Nasser Zouli,
E-mail: nizouli@jazanu.edu.sa

Ayman Yousef,
E-mail: aymanyousef84@gmail.com

Table S1 demonstrates physco-mechanical and thermal characteristics of bricks substituted with different waste material.

No.	Waste material	Compressive strength (MPa)	Water Absorption (%)	Bulk density (Kg/m^3)	Thermal conductivity (W/mK)	References
1.	Diatomaceous earth residues (3 – 10%)	12.7 –9.5	-	1770 - 1670	0.65-0.45	[1]
2.	Rice husk (10 – 30%), wood ash wastes (10 – 30%)	53.4 –13.5 MPa.	21.2% - 32.9%	1839 – 1394	0.68-0.34	[2]
3.	Waste coal (up to 30 %)	–	11.8 – 13.2	1040 - 1250	0.19 - 0.23	[3]
4.	Olive mill waste (0, 5, and 10%)	36.9 – 10.26	14.5% - 32.5%	1920 - 1450	0.638 - 0.436	[4]
5.						
6.	Olive pomace bottom ash (10, 20, 30, 40, and 50%)	33.9 – 10.5	19% - 3 1.5%	1635 - 1278	0.143 -0.166	[5]
7.	Kindling from vine shoots (5, 11, and 17%)	38.04 – 1.556	16.93% - 36.04%	1684 - 1124	0.738 - 0.208	[6]
8.	Wheat straw, olive stone flour (4% and 8%), and sunflower seed cake (4%)	5.3 – 10.9	17.8% - 30%	1700 - 1460	0.55-0.36	[7]
9.	Marble powder (0, 5, 10, 15, 20, 25, 30, and 35%)	34.2 – 8.2	10.9% - 26.9%	2050 - 1590	0.97 -0.40	[8]
10.	Biomass ash (100- 50%), and dust filter (0 – 50%)	17.3 – 5.9	19.8% - 27.5%	1471 - 1346	0.655 - 0. 42	[9]
11.	Glass powder (20 – 35%) and palm oil fly ash (20 – 35%)	15.39 – 7.21	11.48% - 18.5%	1628 – 1338.7	0.39	[10]

12.	Sawdust (5 - 20 vol%), wood ash (5 -15 vol%), and lime mud (5 -15 vol%)	0.83 - 7.56	11.5% - 24%	2080 - 1380	0.55 – 1.12	[11]
13.	Bio - briquette ash (5 – 55%)	3.64 - 4.19	13% - 25%	1470 - 1170	0.65-0.36	[12]
14.	Recycled fine aggregate (25 -100%)	12.75 – 20.98	9.33 - 11.46 %	1968 – 1962	0.82	[13]
15.	Spent Mushroom Materials (0-15 wt%	8.69 – 18.45	13.91– 23.58	1419.98 – 1922.03	0.29 – 0.77	This study

1. Galán-Arboledas, R.J.; Cotes-Palomino, M.T.; Bueno, S.; Martínez-García, C. Evaluation of spent diatomite incorporation in clay based materials for lightweight bricks processing. *Constr. Build. Mater.* **2017**, *144*, 327–337.
2. Eliche-Quesada, D.; Felipe-Sesé, M.A.; López-Pérez, J.A.; Infantes-Molina, A. Characterization and evaluation of rice husk ash and wood ash in sustainable clay matrix bricks. *Ceram. Int.* **2017**, *43*, 463–475.
3. Abdrakhimov, V.Z.; Abdrakhimova, E.S. Promising use of waste coal in the production of insulating material without the use of traditional natural materials. *Inorg. Mater. Appl. Res.* **2017**, *8*, 788–794.
4. Sutcu, M.; Ozturk, S.; Yalamac, E.; Gencel, O. Effect of olive mill waste addition on the properties of porous fired clay bricks using Taguchi method. *J. Environ. Manag.* **2016**, *181*, 185–192.
5. Eliche-Quesada, D.; Leite-Costa, J. Use of bottom ash from olive pomace combustion in the production of eco-friendly fired clay bricks. *Waste Manag.* **2016**, *48*, 323–333.
6. Velasco, P.M.; Ortiz, M.P.M.; Giró, M.A.M.; Melia, D.M.; Rehbein, J.H. Development of sustainable fired clay bricks by adding kindling from vine shoot: Study of thermal and mechanical properties. *Appl. Clay Sci.* **2015**, *107*, 156–164.
7. Bories, C.; Aouba, L.; Vedrenne, E.; Vilarem, G. Fired clay bricks using agricultural biomass wastes: Study and characterization. *Constr. Build. Mater.* **2015**, *91*, 158–163.

8. Sutcu, M.; Alptekin, H.; Erdogmus, E.; Er, Y.; Gencel, O. Characteristics of fired clay bricks with waste marble powder addition as building materials. *Constr. Build. Mater.* **2015**, *82*, 1–8.
9. Sánchez-Martínez, J.; Sesé, F.; Ángel, M.; Infantes-Molina, A. Silica–Calcareous Non Fired Bricks Made of Biomass Ash and Dust Filter from Gases Purification. 2017. Available online: https://reunir.unir.net (accessed on 20 May 2022).
10. Raut, A.N.; Gomez, C.P. Development of thermally efficient fibre-based eco-friendly brick reusing locally available waste materials. *Constr. Build. Mater.* **2017**, *133*, 275–284.
11. Holmes, N.; O'Malley, H.; Cribbin, P.; Mullen, H.; Keane, G. Performance of masonry blocks containing different proportions of incinator bottom ash. *Sustain. Mater. Technol.* **2016**, *8*, 14–19.
12. Sakhare, V.V.; Ralegaonkar, R.V. Use of bio-briquette ash for the development of bricks. *J. Clean. Prod.* **2016**, *112*, 684–689.
13. Ismail, S.; Anas, Z.A.H.; Yaacob, Z. Mechanical and thermal properties of brick produced using recycled fine aggregate. *Key Eng. Mater.* **2016**, *706*, 112–116.
